# Physician Documentation of Social Determinants of Health: Results from Two National Surveys

**DOI:** 10.1007/s11606-024-09184-w

**Published:** 2024-11-18

**Authors:** Bradley E. Iott, Vaishali Patel, Chelsea Richwine

**Affiliations:** 1 Division of General Internal Medicine, Department of Internal Medicine, University of Michigan Medical School, Ann Arbor, MI USA; 2 Office of the Assistant Secretary for Technology Policy, US Department of Health and Human Services, Washington, D.C USA

**Keywords:** Social determinants of health, Electronic health records, Documentation

## Abstract

**Objective:**

We measured physicians’ (1) perceived importance of having access to social determinants of health (SDOH) data received from external providers in the electronic health record (EHR); (2) internal SDOH documentation practices in the EHR, including whether physicians document SDOH in a structured format that may facilitate use; and (3) use of EHR SDOH data to identify community resources and make referrals on behalf of patients.

**Approach:**

We conducted a secondary analysis of two national physician surveys.

**Participants:**

Respondents from the American Board of Family Medicine Recertification Survey (ABFM, *n* = 4040), a survey of family physicians fielded 2021–2022, and the National Physician Health IT Survey (NPHIT, *n* = 3006), a survey of outpatient physicians across specialty areas fielded in 2022.

**Key Results:**

Under half of physicians felt that SDOH data were very important (ABFM: 44.8%, NPHIT: 30.8%). Although most physicians documented SDOH in the EHR (ABFM: 72%, NPHIT: 63.3%), fewer used structured documentation methods (ABFM: 56.3%, NPHIT: 33.2%). In both surveys, physicians who participated in value-based care initiatives, those for whom > 10% of their patient population was considered vulnerable, and those who felt that their clinic had the resources to address patients’ social needs had higher likelihood of documenting SDOH. Sixty-two percent of family physicians reported using SDOH data for identifying resources and making referrals.

**Conclusions:**

In 2022, most physicians documented SDOH data in their EHR, yet fewer used structured methods, limiting data exchange opportunities to address patients’ social needs. Under half of physicians considered access to external SDOH data to be “very” important, suggesting greater reliance on data collected internally and missed opportunities to identify patients who need support. Variation in perceived importance of SDOH data access and SDOH documentation by physician characteristics indicate opportunities to support adoption of structured documentation tools facilitating SDOH data capture and exchange to improve patient-centered care.

**Supplementary Information:**

The online version contains supplementary material available at 10.1007/s11606-024-09184-w.

## INTRODUCTION

There is growing recognition that social determinants of health (SDOH) and social needs play an important role in shaping health outcomes.^1^ The shift towards value-based care initiatives and the use of SDOH data to inform population health interventions have motivated healthcare delivery organizations to screen patients and document SDOH data in electronic health record (EHR) systems.^1,2^ EHR SDOH data may be used to facilitate patient care coordination, including tailoring medical care according to patients’ social situations or offering referrals to community resources to provide more equitable care that enhances patients’ health and well-being.^3^ Beyond supporting patient care, capturing structured SDOH data in the EHR, rather than free-text notes, is critical to supporting downstream uses, such as for community health and population management, and sharing data to connect patients with the resources they need via referrals to community-based organizations.^4–6^

Recently, there has been significant development in data standards for structured SDOH documentation,^7^ including LOINC and ICD-10 Z codes.^8,9^ Despite efforts to enable more structured, systematic collection of SDOH data, a recent study showed that physicians’ rates of documenting and reviewing structured SDOH data were lower than rates of documentation and review of other structured medical, behavioral, and family history domains.^10^ There are several reasons why physicians may be under-documenting SDOH data, or not capturing the information in a structured format that would facilitate exchange and reuse. Physicians’ SDOH documentation practices may be affected by systemic, organizational, and attitudinal factors, including training on why and how to screen; having access to and awareness of, appropriate documentation tools, and access to resources to address social needs.^4,10–14^

Prior work has shown there is variation in physicians’ awareness of SDOH documentation tools, such as screening instruments or templates available in the EHR, which likely affects documentation practices.^13^ How these tools are implemented within organizations may also affect physicians’ documentation and use of these data, as adding SDOH screening, documentation, and referral tasks to existing workflows may create burden for clinicians, potentially contributing to burnout.^15–17^ Finally, physician SDOH documentation may be moderated by views on the relevancy of SDOH to their clinical practice or patient populations.^18^

This study contributes to existing literature on clinicians’ documentation of SDOH by describing physicians’ perspectives on the importance of having access to SDOH data received electronically from other providers (“external” data) in the EHR; physicians’ current SDOH practices, including whether physicians document SDOH in a structured format that would facilitate downstream use; and how these measures vary by clinician and organization characteristics. Finally, we examine whether internal or external EHR SDOH data were used to identify community resources and make referrals on behalf of patients.

## METHODS

### Study Sample

We use two sources of data: (1) the American Board of Family Medicine Recertification Survey (ABFM), a national survey of family physicians, and (2) the National Physician Health IT Survey (NPHIT), a national sample of outpatient physicians across specialty areas.

As further described elsewhere,^19^ the American Board of Family Medicine administers a questionnaire during certification, which has a 100% response rate. Family physicians take this web-based survey on a rolling basis every year based on their initial year of certification, offering a nationally representative sample. The specific survey questions used in this study were developed and revised based on cognitive interviews with family physicians. The survey was fielded from December 12, 2021, to October 12, 2022. A total of 4040 family physicians completed the survey. After answering core questions about practice characteristics and technology use, respondents were randomized to complete one of two modules, one of which contained questions about physicians’ perceived importance of access to SDOH information from other health systems/organizations (*n* = 2034) and the other featured questions about how physicians documented SDOH in the EHR (*n* = 2006, questions shown in Appendix A). We observed no significant differences in physician and practice characteristics between respondents who answered each question module. A separate subsample (*n* = 809, 20% of the overall sample) was asked whether they routinely received comprehensive health assessments that include social concerns (e.g., housing and food security, poverty) and whether they used information on patients’ social concerns to identify needed community resources and make referrals.

The NPHIT was fielded between April and November 2022. Physicians were recruited from a national database (Definitive Healthcare Physician Database). Physicians completed the survey online or via mail (3.6% response rate). Results are weighted for non-response and to generate national estimates. A total of 3006 physicians who provided outpatient care and used an EHR responded to the survey. The same questions about physicians’ perceived importance of access to SDOH information from other health systems/organizations and how physicians documented SDOH in the EHR from the ABFM survey were given to all NPHIT respondents, allowing us to gain insights into physician SDOH documentation practices from these two samples.

### Statistical Analyses

We use logistic regression models to measure associations between physician/practice characteristics and (1) importance of having access to SDOH data in the EHR and (2) use of different SDOH documentation modalities (checkboxes and diagnosis codes) that result in structured data versus free-text clinical notes, and (3) SDOH data utilization among family medicine physicians.

The primary outcome of our first model was a binary variable indicating physician-reported access to external SDOH was very important (1 = very important, 0 = somewhat or not at all important). The primary outcome of our SDOH documentation modality models was binary variables indicating whether the respondent sometimes or often documented SDOH using any modality (free-text note, click button, or diagnosis code) or using a structured modality (click button or diagnosis code, 1 = sometimes or often documented, 0 = never, rarely, or did not know if documented). Our SDOH data use models featured two binary outcome variables indicating whether the respondent (1) routinely received comprehensive health assessments that include social concerns (e.g., housing and food security, poverty) and (2) used information on social concerns of the patient population to identify needed community resources and make referrals (1 = yes, 0 = no or don’t know).

Regression models were adjusted for age, gender, region, rural/urban, principal practice site, practice ownership, principal practice site size, proportion of patient population that was a part of a vulnerable group, EHR vendor, and participation in value-based care initiatives. ABFM models included a covariate indicating if the clinic had resources to address patients’ social needs, while NPHIT models included physician specialty type (primary care, medical, or surgical) and indicators of Medicare and Medicaid/CHIP acceptance. All relationships are reported in terms of predicted probabilities, which offer a useful way to describe the predicted probability of observing outcomes of interest for different values of explanatory variables.^20^

## RESULTS

Nearly half of ABFM respondents (43.7%) and NPHIT respondents (50.1%) practiced in hospital or academic settings (Table 1). Nearly a quarter of both survey respondents (ABFM 23.5%, NPHIT 25.0%) reported that > 50% of their patient population was part of a vulnerable group. Most ABFM physicians (57.7%) felt that their clinic had the resources and tools, such as dedicated staff and linkages to community programs, to address patients’ social needs. Most NPHIT physicians participated in a value-based payment program (61.1%) and most accepted Medicaid/CHIP (80.8%) and Medicare (82.0%).

**Table 1:** Sample Demographics

**Physician, practice, payment, and EHR characteristics**	ABFM (*n* = 4040)		NPHIT (*n* = 3006)	
	# (raw)	% (raw)	# (raw)	% (weighted)
Gender				
Female	2016	49.90	1184	40.63
Male	2024	50.10	1822	59.37
Age				
< 50 years	2417	59.83	892	28.72
50 + years	1623	40.17	2114	71.28
Specialty mix at principal practice				
Primary care/family medicine	4040	100.00	1282	42.65
Medical	-	-	1175	39.09
Surgical	-	-	549	18.26
Principle practice site				
Private practice/urgent care	1073	26.56	866	33.84
FQHC/rural	415	10.27	171	6.58
Academic/hospital/ED/HMO/health system	1766	43.71	1736	50.07
Federal	161	3.99	-	-
Other: family planning, workplace clinic	625	15.47	233	9.51
Role in ownership of principle practice				
No ownership	3005	74.38	2329	73.49
Sole or partial ownership	1035	25.62	580	23.23
Contractor/other	-	-	97	3.27
Principle practice site size				
1–10 clinicians	-	-	1282	42.65
> 11 clinicians	-	-	1724	57.35
Principle practice site size				
Solo practice	358	8.86		
2–5 clinicians	1314	32.52	-	-
6–20 clinicians	1271	31.46	-	-
> 20 clinicians	1097	27.15	-	-
Percentage of patient population at principal practice site is part of a vulnerable group (i.e., uninsured, Medicaid, homeless, low income, non-English speaking, racial/ethnic minority, or otherwise traditionally underserved group)?				
< 10%	1360	33.66	566	19.43
10–49%	1730	42.82	1448	47.39
> 50%	950	23.51	717	24.96
Don’t know	-	-	275	8.21
Does your organization participate in one or more value-based care initiative(s), such as a patient-centered medical home, accountable care organization or pay-for-performance arrangement?				
Yes	2736	67.72	1837	61.14
No	495	12.25	508	19.14
Don’t know	809	20.02	661	19.72
My clinic has the resources and tools, such as dedicated staff and linkages to community programs, to address patients’ social needs				
Agree	2331	57.70	-	-
Neutral	875	21.66	-	-
Disagree	834	20.64	-	-
Accepts Medicaid/CHIP	-	-	2504	80.84
Accepts Medicare	-	-	2520	81.99
Region				
Northeast	577	14.28	651	24.26
Midwest	965	23.89	742	21.48
South	1408	34.85	787	30.58
West	1090	26.98	826	23.67
Location				
Urban	3485	86.26	-	-
Rural	555	13.74	-	-

### Importance of Access to SDOH Information from Other Health Systems/Organizations

**Figure 1:**
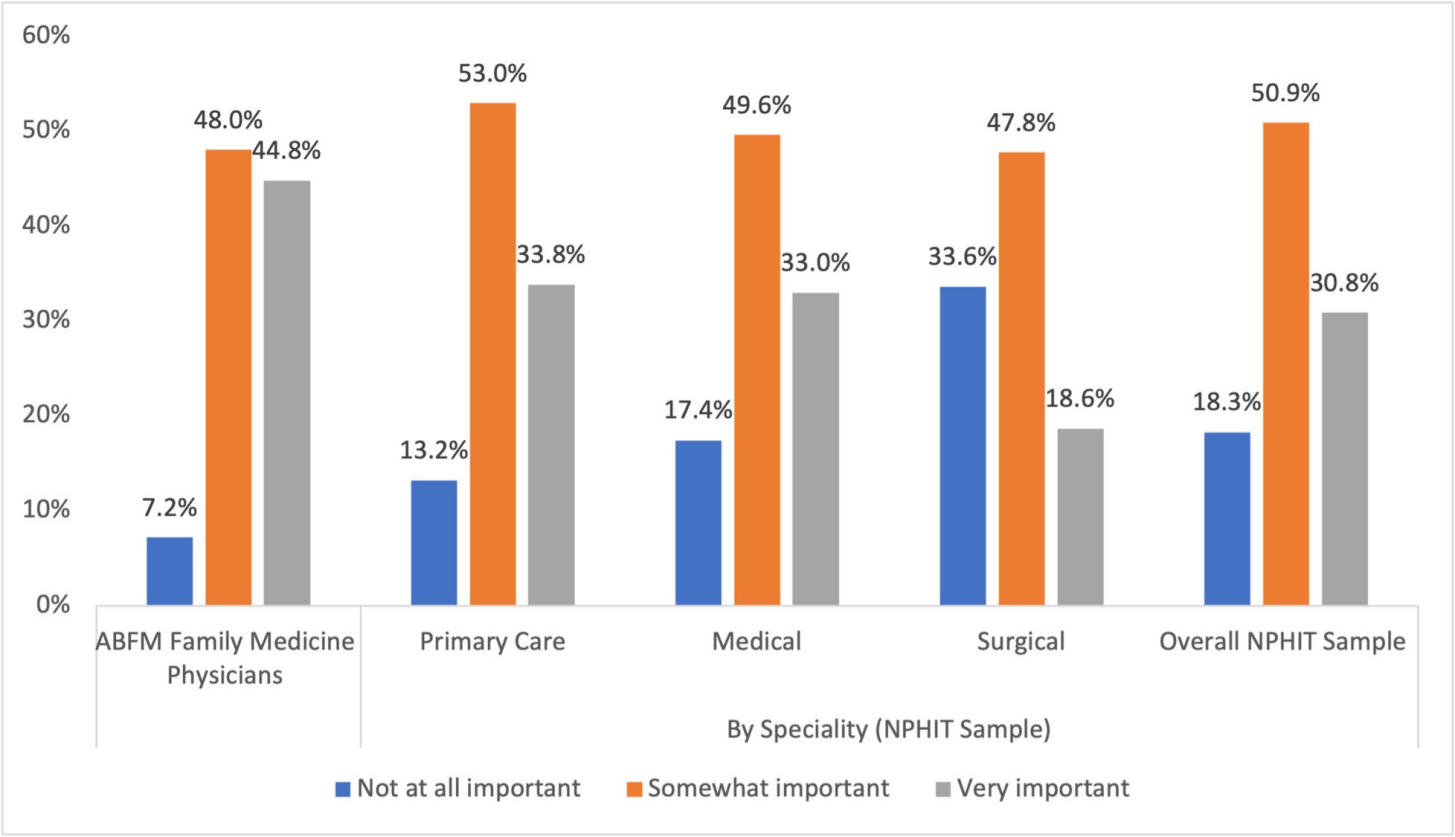
Importance of accessing external SDOH information in the EHR across samples (ABFM, *n* = 2006 and NPHIT, *n* = 3006).

Most respondents in both surveys indicated they believed accessing external SDOH information in the EHR to be somewhat or very important (NPHIT = 81.7%, ABFM = 96.4%, Fig. 1). The number of physicians that endorsed these as “very important” dropped to 44.8% among ABFM survey respondents and 30.8% of the NPHIT respondents. Among NPHIT respondents, this varied by specialty, with rates of indicating external SDOH information as very important being lower among surgeons than primary care physicians and medical specialists (Fig. 1 and Table 2).

In a logistic regression model with ABFM respondents, those who believed that their clinic had the resources and tools to address patients’ social needs had a significantly higher predicted probability of reporting that access to SDOH information was very important (predicted probability = 0.48, *p* = 0.010, Table 2). In a logistic regression model with NPHIT respondents, the predicted probability of physicians reporting that access to external SDOH information was very important was significantly lower among surgical specialists (PP = 0.21, *p* < 0.001) compared to primary care physicians (PP = 0.32) and higher among medical specialists (PP = 0.35), although the difference was not statistically significant. Additionally, NPHIT respondents for whom 50% or more of their patient population was part of a vulnerable group were more likely to value the importance of SDOH data than those with < 10% vulnerable patients (PP = 0.36, *p* < 0.001).

**Table 2:** Logistic Regression Models Predicting Whether Access to External SDOH Information Was Believed to Be Very Important (Predicted Probabilities)

	ABFM (*n* = 2034)^^^	NPHIT (*n* = 3006)^†^
Specialty (Ref: Primary care)		
Primary care	-	0.32
Medical	-	0.35
Surgical	-	0.21***
Participates in value-based care (Ref: No or I don’t know)		
No or I don’t know	0.43	0.29
Yes	0.46	0.32
% of patient population is a part of a vulnerable group (Ref: < 10%)		
< 10%	0.43	0.28
10–49%	0.44	0.30
> 50%	0.49	0.36*
Don’t know	-	0.26
Clinic has the resources to address social needs (Ref: Neutral)		
Neutral	0.41	-
Agree	0.48*	-
Disagree	0.41	-

*p < 0.05, **p < 0.01, ***p < 0.001

^^^Adjusted for practice type, practice ownership, practice size, region, urban/rural setting, EHR, respondent age and gender

^†^Adjusted for practice type, Medicare and Medicaid/CHIP acceptance, practice ownership, practice size, region, EHR, respondent age and gender

### Modalities of SDOH Documentation

**Figure 2:**
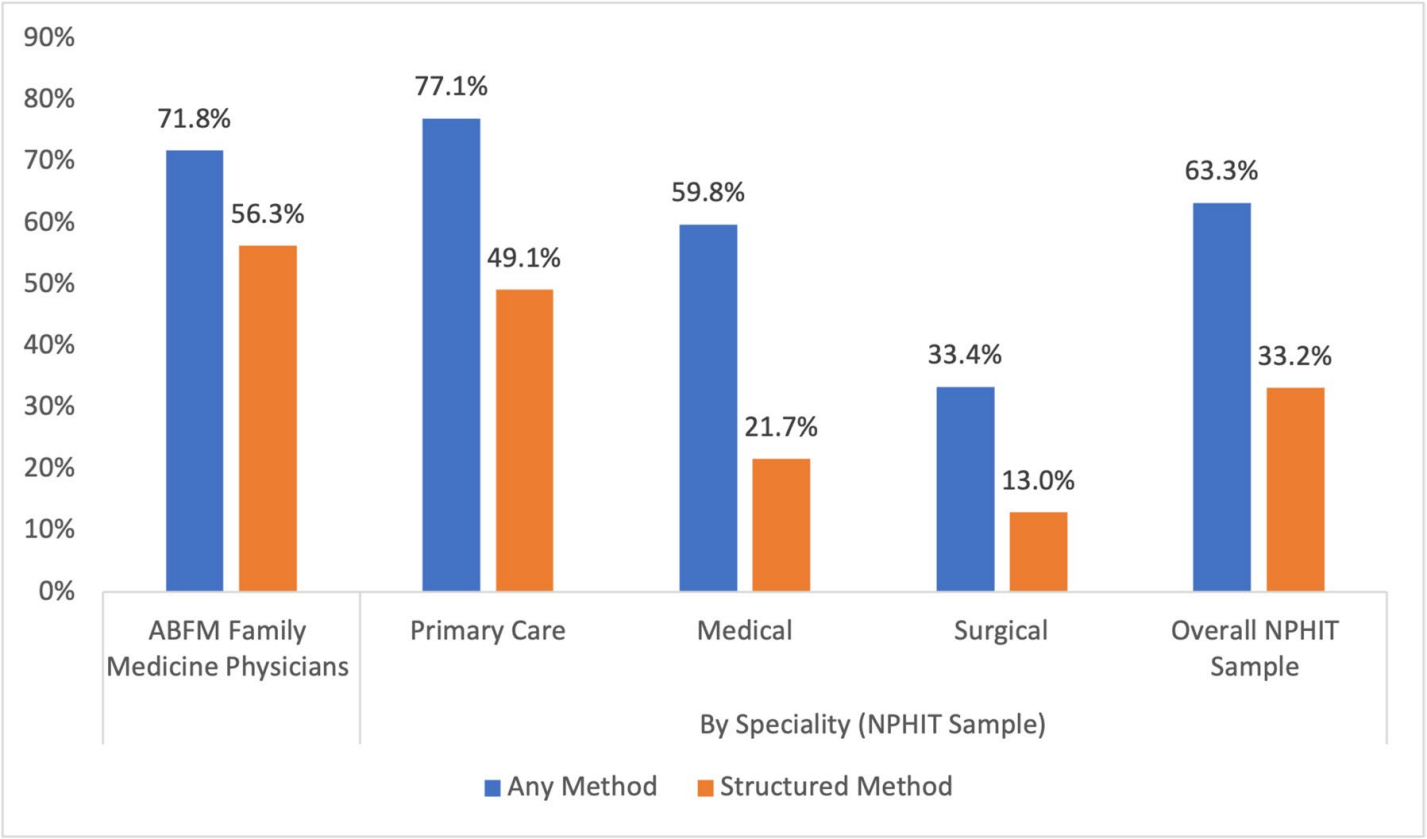
SDOH documentation in the EHR across samples (ABFM, *n* = 2006 and NPHIT, *n* = 3006).

Seventy-two percent of ABFM respondents and 63.3% of NPHIT respondents reported sometimes or often documenting SDOH using at least one of three methods (clinical notes, checkboxes/buttons, or diagnosis codes) to document SDOH in the EHR (Fig. 2). While most respondents in both samples documented SDOH via clinical notes, a higher proportion of ABFM physicians (56.3%) than NPHIT physicians (33.2%) used methods that would result in structured data entry (checkboxes/buttons and diagnosis codes) (Appendix B). Among NPHIT respondents, those who provided primary care were more likely to use structured documentation methods relative to medical care physicians and surgeons (Fig. 2).

Physicians who participated in value-based care had significantly higher likelihood of documenting SDOH via any methods (ABFM: PP = 0.77, *p* < 0.001; NPHIT: PP = 0.67, *p* < 0.001) and structured methods (ABFM: PP = 0.61, *p* < 0.001, NPHIT: PP = 0.38, *p* < 0.001) compared to those not participating. Additionally, among respondents from both surveys, physicians for whom > 10% of their patient population was considered vulnerable had significantly higher likelihood of documenting via any method (ABFM: 10–49% PP = 0.73, *p* = 0.002; > 50%: PP = 0.81, *p* < 0.001; NPHIT: 10–49% PP = 0.66, *p* < 0.001; > 50% PP = 0.71, *p* < 0.001, Table 3). We observed a similar association between patient population vulnerability and use of structured documentation approaches (ABFM: 10–49% PP = 0.57, *p* = 0.009; > 50%: PP = 0.65, *p* < 0.001; NPHIT: 10–49% PP = 0.33, *p* = 0.019; > 50% PP = 0.40, *p* < 0.001).

Among ABFM respondents, those who felt that their clinic had the resources to address patients’ social needs had higher likelihood of documenting via any method (PP = 0.76, *p* = 0.036) or structured methods (PP = 0.61, *p* = 0.027) than those who selected neutral or disagree to that statement. Among NPHIT respondents, medical specialists and surgeons had significantly lower likelihood of using any SDOH documentation methods (PP = 0.62, *p* < 0.001 and PP = 0.40, *p* < 0.001, respectively, Table 3) and structured documentation tools (PP = 0.25, *p* < 0.001 and PP = 0.16, *p* < 0.001, respectively) relative to primary care physicians. Results for regression models predicting the likelihood of using each type of documentation modality are shown in Appendix C.

**Table 3:** Logistic Regression Models Predicting SDOH Documentation (Predicted Probabilities)

	ABFM (*n* = 2006)		NPHIT (*n* = 3006)	
	Documented SDOH using any method^^^	Documented SDOH via structured method (button or diagnosis code)^^^	Documented SDOH using any method^†^	Documented SDOH via structured method (button or diagnosis code)^†^
Specialty (Ref: Primary care)				
Primary care	-	-	0.74	0.45
Medical	-	-	0.62***	0.25***
Surgical	-	-	0.40***	0.16***
Participates in value-based care (Ref: No or I don’t know)				
No or I Don’t Know	0.61	0.47	0.59	0.25
Yes	0.77***	0.61***	0.67***	0.38***
% of patient population is a part of a vulnerable group (Ref: < 10%)				
< 10%	0.65	0.50	0.53	0.27
10–49%	0.73**	0.57**	0.66***	0.33*
> 50%	0.81***	0.65***	0.71***	0.40***
Don’t know	-	-	0.56	0.31
Clinic has the resources to address social needs (Ref: Neutral)				
Neutral	0.61	0.45	-	-
Agree	0.76*	0.61*	-	-
Disagree	0.71**	0.55**	-	-

*p < 0.05, **p < 0.01, ***p < 0.001

^^^Adjusted for practice type, practice ownership, practice size, region, urban/rural setting, EHR, respondent age and gender

^†^Adjusted for practice type, Medicare and Medicaid/CHIP acceptance, practice ownership, practice size, region, EHR, respondent age and gender

### Use of SDOH Data Among Family Medicine Physicians

Approximately 20% of ABFM respondents (*n* = 809) were asked whether they routinely received comprehensive health assessments that include social concerns and whether they used information on patients’ social concerns to identify resources and make referrals. Most ABFM respondents reported receiving assessments with social concerns (71.0%) and 61.8% used information about patients’ social needs to make referrals. The share of respondents who used SDOH data to make referrals for patients was higher among those who documented SDOH using structured methods—78.45% for those using checkboxes/buttons and 77.24% for those using diagnosis codes—compared to respondents using free-text notes (67.45%).

Respondents who believed access to external SDOH information was very important were more likely to use SDOH data to identify resources and make referrals (PP = 0.71, *p* = 0.004). Those who documented SDOH using any method and those who documented SDOH using a structured method were more likely to receive social needs assessments (PP = 0.77, *p* < 0.001 and PP = 0.83, *p* < 0.001, respectively) and to use SDOH data to identify resources and make referrals (PP = 0.66, *p* < 0.001 and PP = 0.73, *p* < 0.001, respectively). Respondents who felt that their clinic had the resources to address social needs, and those who participated in value-based care, had higher likelihood of using this information to identify resources and make referrals to social services for patients (Table 4).

**Table 4:** Logistic Regression Models Predicting How ABFM Respondents Used SDOH Data (Predicted Probabilities, ABFM Subsample, *n* = 809)^†^

	Received social needs assessments	Received social needs assessments	Received social needs assessments	Identified resources and made referrals	Identified resources and made referrals	Identified resources and made referrals
Access to external SDOH information was believed to be very important (Ref: No or I don’t know)						
No or I don’t know	0.68	-	-	0.58	-	-
Yes	0.76	-	-	0.71**	-	-
Documented SDOH using any method (Ref: No)						
No	-	0.55	-	-	0.45	-
Yes	-	0.77***	-	-	0.66***	-
Documented SDOH via structured method (button or diagnosis code) (Ref: No)						
No	-	-	0.58	-	-	0.46
Yes	-	-	0.83***	-	-	0.73***
Clinic has the resources to address social needs (Ref: Neutral)						
Neutral	0.68	0.65	0.67	0.56	0.49	0.51
Agree	0.78	0.76*	0.76	0.73**	0.69***	0.68**
Disagree	0.58	0.61	0.61	0.46	0.49	0.49
Participates in value-based care (Ref: No or I don’t know)						
No or I don’t know	0.66	0.61	0.62	0.45	0.47	0.48
Yes	0.74	0.74**	0.74**	0.72***	0.66**	0.65**
% of patient population is a part of a vulnerable group (Ref: < 10%)						
< 10%	0.71	0.70	0.70	0.58	0.63	0.63
10–49%	0.72	0.73	0.73	0.64	0.60	0.59
> 50%	0.73	0.65	0.66	0.73*	0.56	0.58

*p < 0.05, **p < 0.01, ***p < 0.001

^†^Adjusted for practice type, practice ownership, practice size, region, urban/rural setting, EHR, respondent age and gender

## DISCUSSION

In two national surveys, we found that while most physicians—particularly primary care physicians—indicated that it was somewhat or very important to be able to view external SDOH information in the EHR, fewer physicians felt that having access to SDOH data was very important. The perceived value of SDOH varied by specialty, with fewer surgical specialists rating access to external SDOH data as very important. EHR SDOH documentation rates were relatively high among physicians overall, especially among family medicine physicians. However, rates of documentation using structured methods were lower, with about one-third of physicians and about half of family medicine physicians reporting using structured methods. Those working in medical or surgical specialties were less likely to document overall and using structured methods than primary care physicians.

Variation in the documentation and use of SDOH data by specialty may be due, in part, to differences in perceived importance of accessing external SDOH data in the EHR which suggests there are opportunities to promote structured documentation and use of SDOH data in a variety of clinical settings. For instance, lower perceived importance of external SDOH data among surgeons relative to other types of physicians contrasts with known associations between SDOH and post-operative outcomes.^21^ While some SDOH screening and referral interventions occur in surgical settings,^22,23^ many current efforts are focused in primary care settings.^24^ Given that SDOH may play a role in post-operative outcomes, educating surgeons regarding the value of SDOH data in surgical and specialty settings may be beneficial. Future work should assess capacity for and interest in providing screening and referrals in specialty settings. In interpreting these findings, it should be noted that other members of the healthcare team, including community health workers, nurses, care coordinators, case managers, and social workers, may be more likely to conduct SDOH screening and offering resources to address social needs than physicians.^25,26^ This may be due to workflow design, including having more time with patients or conducting screening during the rooming process. However, physicians play an important role in using SDOH data to adjust medical care decisions to accommodate social circumstances and to offer resources to address social needs.^3^

Not surprisingly, physicians who had a higher proportion of vulnerable patients were more likely to indicate that access to external SDOH data was very important. We also show that family medicine physicians who reported having the necessary resources in their clinics to address social needs were more likely to believe that access to external SDOH data was very important. Furthermore, perceptions regarding having the necessary resources to address social needs were also associated with greater collection and use of SDOH data, and identifying resources and making referrals. Having in-clinic resources for screening and referrals, education about the impact of SDOH on health, and training on how to address social needs may be important facilitators of conducting screening and using that information to inform clinical decision-making and to make referrals.^15^ Those serving high proportions of marginalized patients may practice in resource-constrained environments, and thus, attention must be paid to ensure that patients have access to screening and referral resources regardless of where they seek care.^27^ Physicians who serve vulnerable populations may have limited access to resources to address social needs, which in turn may perpetuate existing inequities.^27^

Sharable structured SDOH data helps to ensure physicians have access to the timely and accurate data about patients’ needs. Recent studies have demonstrated that EHR SDOH documentation may underestimate the prevalence of SDOH.^4,28^ We affirm prior work indicating that documentation of SDOH occurs frequently via free-text notes, while the use of structured data capture tools remains limited.^6^ In addition to primary care, those practicing in clinics engaged in value-based care or served a higher proportion of vulnerable groups were more likely to document using a structured method. Variation in SDOH documentation practices suggests the need for outreach to increase SDOH documentation rates among certain physician populations, such as specialists, as SDOH documentation has been shown to be important for care coordination^29,30^ and to support efforts to assist patients with social needs or adjust medical care decisions based on social circumstances.^3^ Additionally, there are several standards in development which will require SDOH screening and referrals to address social needs, which may increase adoption of structured SDOH EHR documentation approaches.^31^

Efforts to increase structured SDOH documentation will be important to facilitate exchange and use of SDOH data.^31,32^^(p19)^ Value-based payment models and incentives are beginning to encourage the collection and use of these data,^32^^(p19)^ and starting in 2024, Medicare is requiring screening of all inpatients for food insecurity, housing instability, transportation needs, utility difficulties, and interpersonal safety.^33,34^ As more patients are regularly screened for SDOH, structured SDOH documentation will be important for quality measurement and reporting, and indeed, several SDOH-focused quality measures under development will require reporting on the prevalence of SDOH screening.^31,35–40^ Additionally, structured SDOH data may be used in clinical decision support algorithms to support tailoring medical decisions based on patients’ social needs^41^ and in community resource referral platforms to facilitate referrals to local resources.^42^ The increased prevalence of SDOH screening, and subsequent documentation of SDOH in the EHR, spurred by the new Medicare inpatient screening requirement further underscores the need to support all types of physicians on healthcare teams with resources to best make use of these data to support patients.

Our findings suggest there is opportunity to improve structured data collection and use of SDOH data to address social needs. In recognition of the urgent need to create a more integrated health and social care system that can effectively meet patients’ needs and improve equitable opportunities for attaining health and well-being, the U.S. Department of Health and Human Services recently released a Call to Action urging clinicians—including primary care physicians and specialists—to consistently screen and identify patients with social needs using diagnosis codes, and to leverage partnerships with community-based organizations and community care hubs that can help facilitate care coordination and service delivery to address social needs.^43^ Further research is necessary to determine how best to support clinicians as reporting on SDOH screening and referrals become increasingly required or strongly encouraged through financial incentives.^31^

This study has several limitations that must be considered. First, all included physician documentation measures relied on respondent self-report and may not reflect all documentation efforts in each clinic. Furthermore, these measures do not provide indication of frequency nor volume of EHR SDOH documentation or methods used. While the survey provides insight into the different types of methods used for SDOH documentation, it is unclear how often each of these methods is used for documentation.

We show that while many physicians value having access to SDOH data from outside sources, fewer physicians consider these external data to be “very” important. While most physicians document SDOH data, yet fewer use structured methods, limiting opportunities for exchange or use of these data to connect patients to resources. Physicians’ perceived value and documentation practices varied by specialty, availability of resources to address social needs, participation in value-based care, and patient population vulnerability, suggesting there are myriad factors potentially affecting the documentation and use of SDOH data. As efforts are underway to develop tools to help inform medical care decisions and provide services to address social needs, it is critical to ensure physicians have access to complete and accurate EHR SDOH data and have the resources they need to effectively use data captured through screening.

## Supplementary Information

Below is the link to the electronic supplementary material.

Supplementary file1 (DOCX 16 KB)

## Data Availability

The data underlying this article cannot be shared publicly in order to protect the privacy of individuals represented in the dataset.
