# Supplementary material for: Physician Documentation of Social Determinants of Health: Results from Two National Surveys
Source: J Gen Intern Med. 2024 Nov 18;40(3):620–8. doi: 10.1007/s11606-024-09184-w (PMC11861457; doi:10.1007/s11606-024-09184-w)
Supplement: Supplementary file 1 — Supplementary file1 (DOCX 16 KB) [file 11606_2024_9184_MOESM1_ESM.docx]

**Supplementary Information**

Appendix A. Survey Questions

| Outcome | Question | Response Options |
| --- | --- | --- |
| Importance of Accessing External SDOH Information in the EHR | Access to social determinants of health information (e.g. housing stability, food insecurity) from other health systems/organizations | Not at all Important, Somewhat Important, Very Important |
| SDOH Documentation via Box/Button | How often do you document screening for social needs (such as transportation, housing, food insecurity) in your primary outpatient EHR by checking a box/button within the EHR? | Never, Rarely, Sometimes, Often, Don’t Know |
| SDOH Documentation via Free Text Note | How often do you document screening for social needs (such as transportation, housing, food insecurity) in your primary outpatient EHR by writing it in a note? | Never, Rarely, Sometimes, Often, Don’t Know |
| SDOH Documentation via Diagnosis Code | How often do you document screening for social needs (such as transportation, housing, food insecurity) in your primary outpatient EHR by entering it as a diagnosis (i.e. ICD-10-CM Z codes)? | Never, Rarely, Sometimes, Often, Don’t Know |
| Received Social Needs Assessments | Routinely receive comprehensive health assessments that include social concerns (e.g., housing and food security, poverty, etc) | Yes, No, Don’t Know |
| Identified Resources and Made Referrals | Using information on social concerns of the patient population to identify needed community resources and make referrals | Yes, No, Don’t Know |

Appendix B. SDOH Documentation Modalities across NPHIT and ABFM Samples (NPHIT, n=3,006, and ABFM, n=2,006)


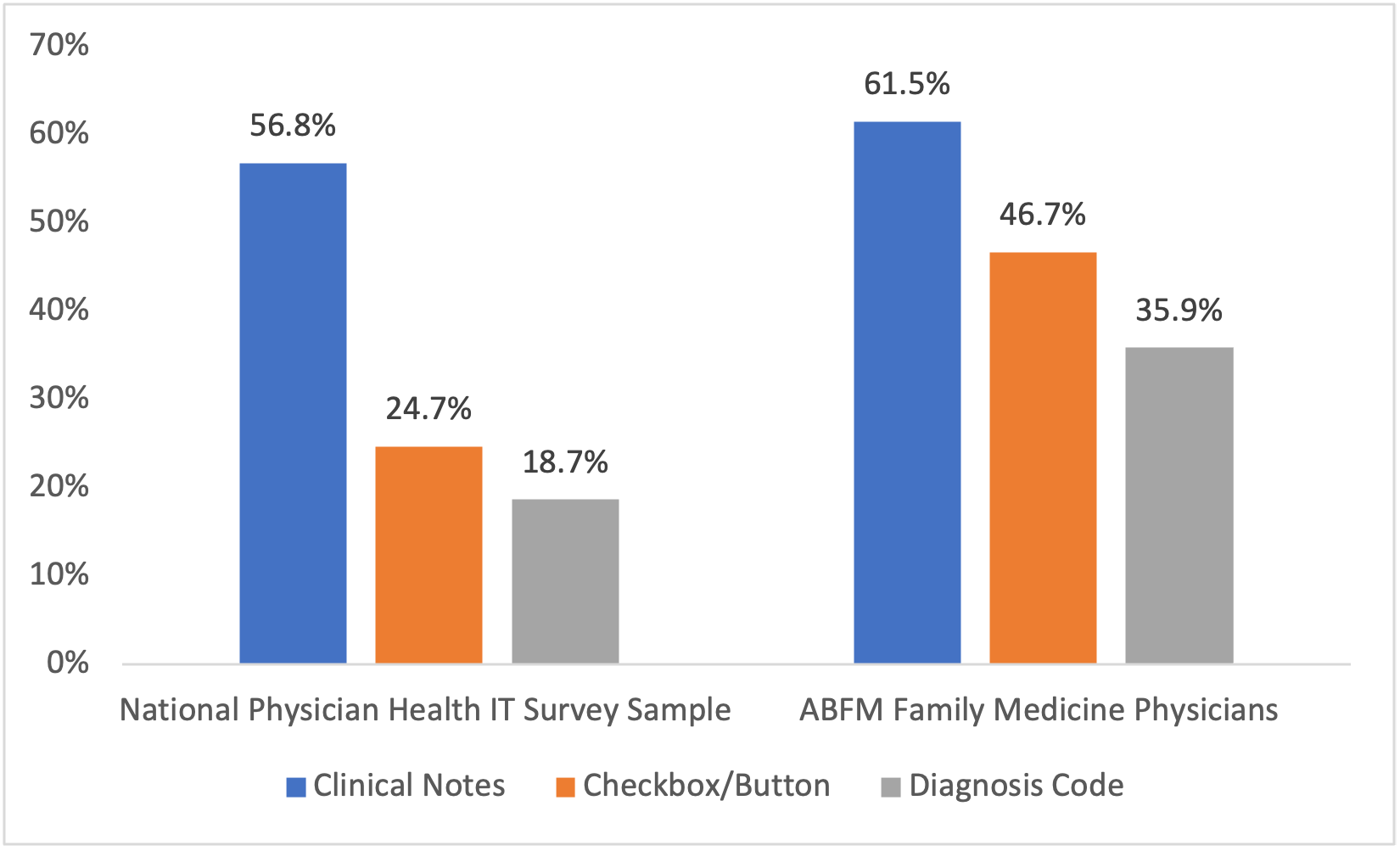


Appendix C. Logistic Regression Models Predicting SDOH Documentation Modality (Predicted Probabilities)

|  | ABFM (n=2,006) | | | NPHIT (n=3,006) | | |
| --- | --- | --- | --- | --- | --- | --- |
|  | Documented SDOH via Button† | Documented SDOH via Button† | Documented SDOH via Button† | Documented SDOH via Button† | Documented SDOH via Diagnosis Code† | Documented SDOH via Note† |
| Specialty (Ref: Primary Care) |  |  |  | 0.34 | 0.27 | 0.64 |
| Medical | - | - | - | 0.17*** | 0.12*** | 0.57** |
| Surgical | - | - | - | 0.12*** | 0.08*** | 0.35*** |
| Participates in Value-Based Care (Ref: No or I don't know) |  |  |  |  |  |  |
| Yes | 0.52*** | 0.38** | 0.65*** | 0.29*** | 0.21*** | 0.59** |
| No | 0.36 | 0.32 | 0.53 | 0.17 | 0.14 | 0.53 |
| % of Patient Population is a part of a Vulnerable Group (Ref: <10%) |  |  |  |  |  |  |
| <10% | 0.42 | 0.30 | 0.53 | 0.22 | 0.12 | 0.46 |
| 10-49% | 0.47 | 0.36** | 0.63*** | 0.24 | 0.19** | 0.59*** |
| >50% | 0.54*** | 0.45*** | 0.71*** | 0.29* | 0.23*** | 0.65*** |
| Don’t Know |  |  |  | 0.23 | 0.19 | 0.47 |
| Clinic has the resources to address social needs (Ref: Neutral) |  |  |  |  |  |  |
| Agree | 0.51 | 0.39 | 0.66** | - | - | - |
| Neutral | 0.46 | 0.35 | 0.58 | - | - | - |
| Disagree | 0.35** | 0.28* | 0.53 | - | - | - |

* p < 0.05, ** p < 0.01, *** p < 0.001

^^^ Adjusted for practice type, practice ownership, practice size, region, urban/rural setting, respondent age and gender.

^†^ Adjusted for practice type, Medicare and Medicaid/CHIP acceptance, practice ownership, practice size, region, EHR, respondent age and gender.
